# A Bioinformatics Analysis Identifies the Telomerase Inhibitor MST-312 for Treating High-STMN1-Expressing Hepatocellular Carcinoma

**DOI:** 10.3390/jpm11050332

**Published:** 2021-04-22

**Authors:** Szu-Jen Wang, Pei-Ming Yang

**Affiliations:** 1 Division of Gastroenterology, Department of Internal Medicine, Yuan’s General Hospital, Kaohsiung 80249, Taiwan; ezpperqoo@gmail.com; 2 Graduate Institute of Cancer Biology and Drug Discovery, College of Medical Science and Technology, Taipei Medical University, Taipei 11031, Taiwan; 3 Program for Cancer Molecular Biology and Drug Discovery, College of Medical Science and Technology, Taipei Medical University, Taipei 11031, Taiwan; 4 Cancer Center, Wan Fang Hospital, Taipei Medical University, Taipei 11696, Taiwan; 5 TMU Research Center of Cancer Translational Medicine, Taipei Medical University, Taipei 11031, Taiwan

**Keywords:** bioinformatics, cancer genomics, cell cycle, hepatocellular carcinoma, stathmin 1

## Abstract

Hepatocellular carcinoma (HCC) is a relatively chemo-resistant tumor. Several multi-kinase inhibitors have been approved for treating advanced HCC. However, most HCC patients are highly refractory to these drugs. Therefore, the development of more effective therapies for advanced HCC patients is urgently needed. Stathmin 1 (STMN1) is an oncoprotein that destabilizes microtubules and promotes cancer cell migration and invasion. In this study, cancer genomics data mining identified STMN1 as a prognosis biomarker and a therapeutic target for HCC. Co-expressed gene analysis indicated that STMN1 expression was positively associated with cell-cycle-related gene expression. Chemical sensitivity profiling of HCC cell lines suggested that High-STMN1-expressing HCC cells were the most sensitive to MST-312 (a telomerase inhibitor). Drug–gene connectivity mapping supported that MST-312 reversed the STMN1-co-expressed gene signature (especially BUB1B, MCM2/5/6, and TTK genes). In vitro experiments validated that MST-312 inhibited HCC cell viability and related protein expression (STMN1, BUB1B, and MCM5). In addition, overexpression of STMN1 enhanced the anticancer activity of MST-312 in HCC cells. Therefore, MST-312 can be used for treating STMN1-high expression HCC.

## 1. Introduction

Hepatocellular carcinoma (HCC) is one of the major causes of cancer-associated death in the world [1]. The main curative treatments for HCC are surgical resection and liver transplantation, which only benefits 15–25% of HCC patients. In addition, there is no reliably effective therapy for advanced or metastatic HCC patients [2,3]. Molecular targeted agents have been viewed as new treatment options, such as multi-kinase inhibitors, sorafenib, regorafenib, and lenvatinib [4,5,6]. However, these drugs only provide a short increase of median overall survival in HCC patients [4,5,6,7]. Thus, there is an urgent need to design more effective therapeutic strategies for HCC.

Stathmin 1, encoded by the human *STMN1* gene, was first cloned in 1989 [8]. STMN1 is a cytosolic phosphoprotein that regulates microtubule dynamics by promoting microtubule destabilization and preventing tubulin polymerization [9]. Mechanistically, the unphosphorylated form of STMN1 (through the stathmin-like domain) interacts with two molecules of dimeric α/β-tubulin to form a tight ternary complex called the T2S complex, thereby limiting the availability of free tubulins [10,11]. In contrast, phosphorylation of STMN1 on multiple serine residues (Ser16, Ser25, Ser38, and Ser63) reduces its microtubule-destabilizing activity [12].

STMN1 is also known as Oncoprotein 18 (Op18). Increased STMN1 expression has been observed in numerous human tumors including HCC, which is associated with aggressive tumor phenotypes and poor prognosis [13,14,15,16,17]. Therefore, STMN1 has been viewed as a therapeutic target for cancer treatment [18,19,20]. In HCC, the anticancer effects of the lentivirus-mediated RNA interference (RNAi) targeting enhancer of zeste homolog 2 (EZH2), gambogic acid, and thyroid hormone are found through downregulation of STMN1 [21,22,23]. In addition, STMN1 expression can determine the sensitivity to apoptosis in HCC cells during hepatitis C viral (HCV) replication [24]. The oncogenic mechanism of STMN1 overexpression is largely dependent on its ability to destabilize microtubules, leading to the promotion of cancer cell division, migration, and invasion [9,25].

In this study, we employed bioinformatics approaches and identified MST-312, a telomerase inhibitor, as an effective treatment for high-STMN1-expressing HCC. Mechanistically, MST-312 could reverse the co-expressed gene network that was related to cell cycle alteration in HCC. MST-312 may serve as a precision treatment for HCC in the future.

## 2. Materials and Methods

### 2.1. Cancer Genomics Data Mining

The cancer genomics data in HCC were analyzed on the cBioPortal and GEPIA websites (https://www.cbioportal.org/ and http://gepia2.cancer-pku.cn/, respectively; accessed on: 29 July 2019) [26,27,28]. For cBioPortal analysis, the liver hepatocellular carcinoma (LIHC) PanCancer Atlas dataset from The Cancer Genome Atlas (TCGA) was used [29]. Complete samples (*n* = 348) with mutation, copy number alteration (CNA), and mRNA expression data were used for cBioPortal data mining. For GEPIA data mining, tumors (*n* = 369) and the matched normal (*n* = 50) samples in LIHC dataset were considered. Kaplan–Meier survival plots were also created using the cBioPortal and GEPIA databases. Three microarray datasets containing the normal and HCC tissues were analyzed on the Oncomine database (https://www.oncomine.org/; accessed on: 29 July 2019), including Chen Liver (normal = 76 and HCC = 104) [30], Roessler Liver 2 (normal = 220 and HCC = 225) [31], and Wurmbach Liver (normal = 10, cirrhosis = 13, dysplasia = 17, and HCC = 35) [32].

### 2.2. Kyoto Encyclopedia of Genes and Genomes (KEGG) Pathway Analysis

The *STMN1*-co-expressed genes with a correlation score of more than 0.7 were obtained from the GEPIA and Oncomine websites (Table S1). KEGG pathway enrichment was conducted using the WebGestalt online tool [33] at http://www.webgestalt.org/ (accessed on: 29 July 2019) or the compareCluster function in the clusterProfiler R-package [34].

### 2.3. Cancer Drug Sensitivity Analysis

The relationship between *STMN1* mRNA expression and drug sensitivity was obtained from the Cancer Therapeutics Response Portal (CTRP) database [35,36,37] at https://portals.broadinstitute.org/ctrp/ (accessed on: 29 July 2019). The correlation between *STMN1* mRNA expression and MST-312 drug activity was visualized using the CellMinerCDB online tool [38] at https://discover.nci.nih.gov/cellminercdb/ (accessed on: 29 July 2019). The correlation between MST-312 drug activity from the area under the curve (AUC) and *STMN1* gene dependency based on clustered regularly interspaced short palindromic repeat (CRISPR) screening was obtained from the DepMap online tool [39] at https://depmap.org/ (accessed on: 29 July 2019). Lower AUC values indicate higher drug activity.

### 2.4. Connectivity Map (CMap) Analysis

The CMap analysis was performed online via https://clue.io/ (accessed on: 9 April 2021) [40]. The 57 *STMN1*-co-expressed genes were queried for drugs and gene knockdowns that could reverse the queried gene signature. The “Touchstone” tool was used to explore the relationship between MST-312 and gene knockdowns. The results were interpreted from the connectivity scores (from −100 to 100). Positive or negative scores indicate similarity or dissimilarity between two gene signatures, respectively.

### 2.5. Cell Culture, Stable Transfection, and Cell Viability Assay

The human hepatocellular carcinoma (HCC) cells, PLC/PRF/5 (PLC5) and HepG2, were purchased from the Bioresource Collection and Research Center (BCRC) of the Food Industry Research and Development Institute (Hsinchu, Taiwan). Cells were maintained in Dulbecco’s Modified Eagle’s Medium (DMEM) containing 10% fetal bovine serum and cultured in a 37 °C humidified incubator with 5% CO_2_. For the establishment of STMN1-overexpressing clones, PLC5 and HepG2 cells were transfected with p-EGFP-STMN1 or its control vector (p-EGFP) and then selected with 1 mg/mL G418 for at least 3 months. The STMN1-EGFP plasmid was a gift from Lynne Cassimeris (Addgene plasmid #86782; http://n2t.net/addgene:86782 accessed on: 29 July 2019; RRID: Addgene_86782) [41]. The cell viability was determined with Alamar Blue reagent (Thermo Fisher Scientific, Waltham, MA, USA) according to the manufacturer’s instruction.

### 2.6. Western Blotting

Western blotting was performed as described previously [42]. STMN1 (GTX104707 and GTX113341), MCM5 (GTX114090), BUB1B (GTX111289), and GAPDH (GTX100118) antibodies were purchased from GeneTex (Hsinchu, Taiwan). The PARP1 antibody (#9542) was purchased from Cell Signaling Technologies (Beverly, MA, USA). The caspase 3 antibody (19677-1-AP) was purchased from ProteinTech Group (Chicago, IL, USA).

## 3. Results

### 3.1. STMN1 Overexpression Is Associated with Poorer Prognosis in HCC

**Figure 1:**
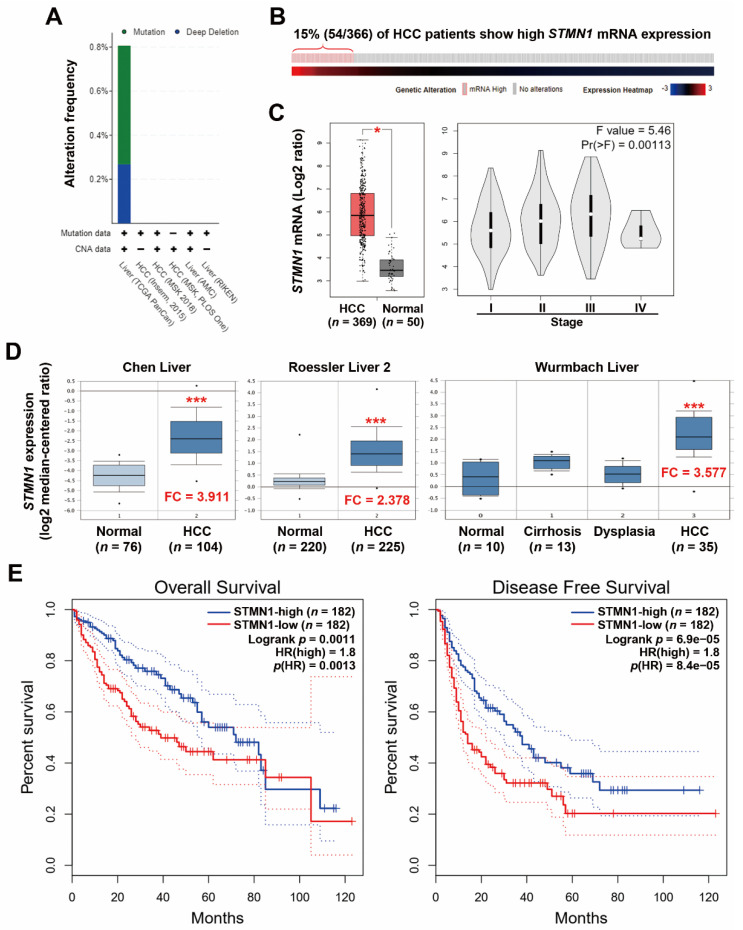
STMN1 overexpression was associated with poor prognosis in HCC: (**A**) Genetic alterations of the *STMN1* gene in five HCC datasets were analyzed through the cBioPortal database. CNA, copy number alteration. The “+” symbol indicates that the used dataset contains mutation or CNA data; (**B**) A bar code plot (OncoPrint) for *STMN1* mRNA expression in HCC (TCGA-LIHC, PanCancer Atlas). The cases highlighted in red grids (mRNA high) had mRNA expression z-score higher than 1. The mRNA expression z-score means the relative expression of a gene in a tumor sample to the gene’s expression distribution in a reference population (diploid tumor samples); (**C**) The *STMN1* mRNA expressions in normal and cancerous liver tissues (the left part) and in different tumor stages (the right part) were analyzed through the GEPIA website (TCGA-LIHC). * *p* < 0.05 compared with the normal group using one-way ANOVA; (**D**) The *STMN1* mRNA expression in normal and cancerous liver tissues in three cohorts was analyzed through the Oncomine database. *** *p* < 0.01 compared with the normal group using the Student’s t-test. FC, fold change; (**E**) The impact of *STMN1* mRNA expression on the overall and disease-free survival of HCC patients was analyzed through the GEPIA website. The group cutoff value was the median *STMN1* mRNA expression.

In recent years, increasing large-scale cancer genomics data and related analytic tools have become publicly available, making it possible to re-evaluate the role of a specific gene in cancers. Although *STMN1* has been cloned since 1989 [8], investigation of its role in HCC is still limited. In this study, we queried the HCC cancer genomics data via the cBioPortal website to determine the genetic alterations (mutations and copy number variations) of the *STMN1* gene in HCC. As shown in Figure 1A, only 2 of 372 (0.54%) and 1 of 372 (0.27%) HCC patients harbored *STMN1* gene mutation and deep deletion, respectively, in the TCGA-LIHC dataset, and no alteration was found in the other five datasets. The *STMN1* mRNA expression in HCC was further investigated using the TCGA-LIHC dataset (Figure 1B). We found that 54 of 366 (15%) HCC patients had higher *STMN1* mRNA expression. When compared with the adjacent normal tissues, HCC tumor tissues indeed displayed higher *STMN1* mRNA expression (Figure 1C, the left part). In addition, a stage-dependent increase of *STMN1* mRNA in HCC tissues was found (Figure 1C, the right part).

To confirm the above observation, microarray gene expression profiles of normal and HCC tissues were obtained from the Oncomine database to compare *STMN1* mRNA expression. As shown in Figure 1D, *STMN1* mRNA was frequently overexpressed in HCC tissues in three microarray datasets. In addition, a slight increase of *STMN1* mRNA was found in precancerous liver tissues (cirrhosis and dysplasia), suggesting that STMN1 may play an early role during hepatocarcinogenesis. Previous studies consistently suggest that protumorigenic overexpression of STMN1 is associated with hepatocarcinogenesis [16,43,44].

Previous studies have shown that STMN1 is frequently overexpressed in HCC, which is associated with tumor progression, early recurrence, and poor prognosis [15,16,17,45]. To ascertain the prognostic impact of STMN1 overexpression, Kaplan–Meier survival plots for overall and disease-free survival in HCC patients (TCGA-LIHC dataset) with higher and lower *STMN1* mRNA expression were created using the GEPIA web-based tool. As shown in Figure 1E, HCC patients with high *STMN1* mRNA expression had poorer overall and disease-free survival. Similarly, the top 15% of HCC patients with higher *STMN1* mRNA expression (Figure 1B) also had lower overall and disease-free survival (Figure S1).

### 3.2. STMN1 Co-Expresses with Genes Related to Cell Cycle Regulation

**Figure 2:**
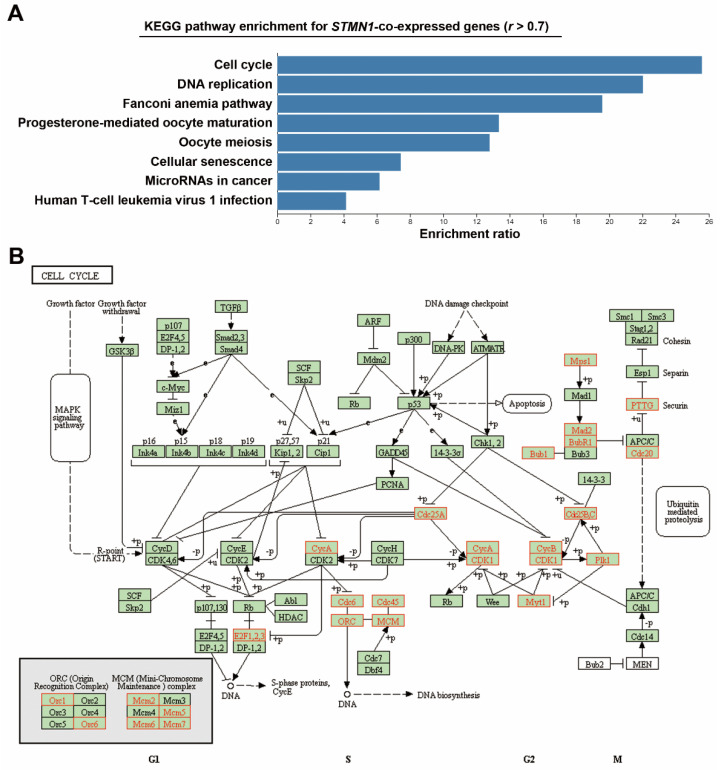
Co-expression of STMN1 with cell-cycle regulatory genes in HCC (TCGA-LIHC): (**A**) *STMN1*-co-expressed genes were analyzed by GSEA using the WebGestalt web tool; (**B**) *STMN1*-co-expressed genes were mapped with KEGG cell cycle (hsa04110) pathway using the WebGestalt web tool. The mapped genes are highlighted in red.

To investigate the impact of STMN1 overexpression, *STMN1*-co-expressed genes were retrieved from the TCGA-LIHC dataset via the cBioPortal website (Table S1). KEGG pathway enrichment for these genes was performed using the WebGestalt online tool. As shown in Figure 2A, STMN1 overexpression was correlated with pathways related to cell cycle regulation such as cell cycle, DNA replication, oocyte maturation/meiosis, and cellular senescence. The KEGG cell cycle (hsa04110) pathway was mapped to *STMN1*-co-repressed genes as a representative (Figure 2B). We found that components for DNA replication (*CDC6*, *CDC45*, *ORC1*/*6*, *MCM2*/*5*/*6*/*7*), G2/M transition (*CDC25A*/*B*/*C*, *CDK1*, *PLK1*, *Cyclins A*/*B*), and mitosis (*TTK*/*MPS1*, *MAD2*, *BUB1B*/*BUBR1*, *BUB1*, *PTTG1*, *CDC20*) were upregulated, suggesting the active cell proliferation rate in high-*STMN1*-expressing HCC cells.

**Figure 3:**
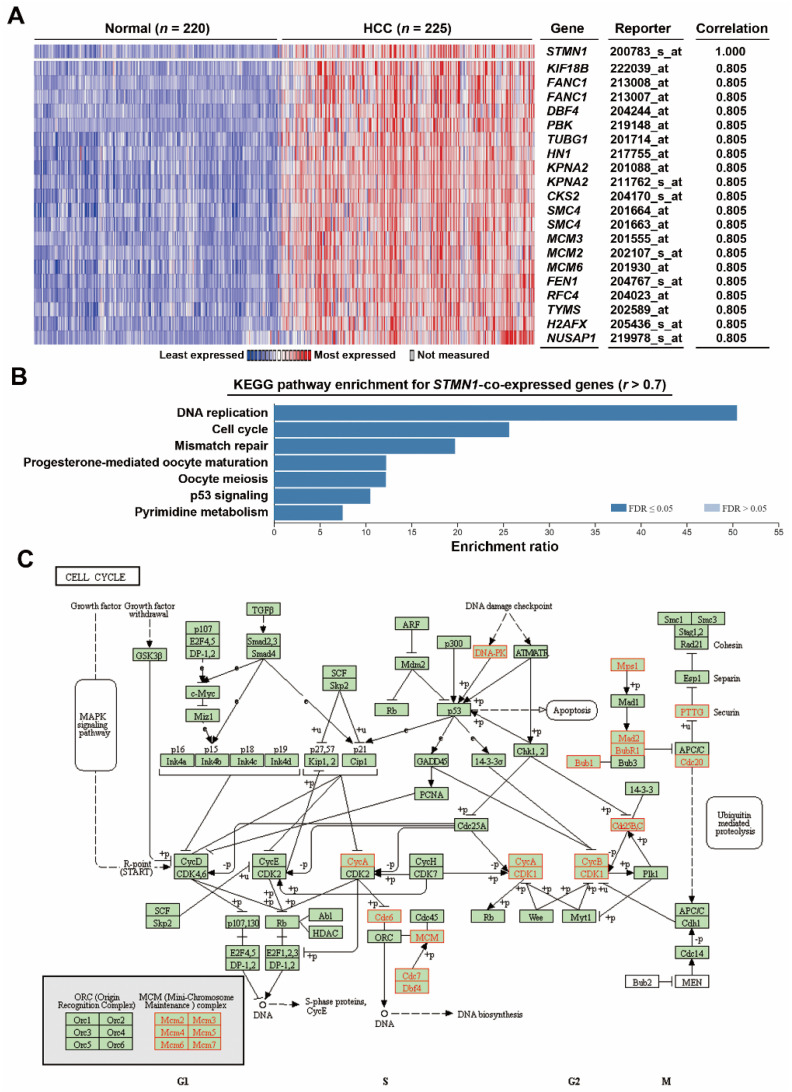
Co-expression of STMN1 with cell-cycle regulatory genes in HCC (Oncomine): (**A**) Genes co-expressed with *STMN1* were obtained from the Oncomine database (Roessler Liver 2). This heat map showed the related expression levels for the top 20 genes with *STMN1* gene; (**B**) *STMN1*-co-expressed genes were analyzed by GSEA using the WebGestalt web tool; (**C**) *STMN1*-co-expressed genes were mapped with KEGG cell cycle (hsa04110) pathway using the WebGestalt web tool. The mapped genes are highlighted in red.

To confirm the above analysis, an additional microarray dataset (Roessler Liver 2) containing 220 normal tissues and 225 HCC tissues was used to prepare *STMN1*-co-expressed genes via the Oncomine database (Table S1). The heat map for the top 20 co-expressed genes is shown in Figure 3A. Similarly, cell cycle and DNA replication pathways were the most enriched pathways (Figure 3B). KEGG cell cycle (hsa04110) mapping (Figure 3C) also showed the upregulation of genes related to DNA replication (*CDC6*, *MCM2*/*3*/*4*/*5*/*6*/*7*, *CDC7*, *DBF4*), G2/M transition (*CDC25B*/*C*, *CDK1*, *Cyclins A*/*B*), and mitosis (*TTK*/*MPS1*, *MAD2*, *BUB1B*/*BUBR1*, *BUB1*, *PTTG1*, *CDC20*). Consistently, a cross-comparison of KEGG pathway enrichment among four datasets (TCGA-LIHC, Chen Liver, Roessler Liver 2, and Wurmbach Liver) showed that cell-cycle-related genes were the most common genes co-expressed with *STMN1* in HCC (Figure S2).

### 3.3. HCC Cells with Higher STMN1 Expression Are Sensitive to A Telomerase Inhibitor MST-312

**Figure 4:**
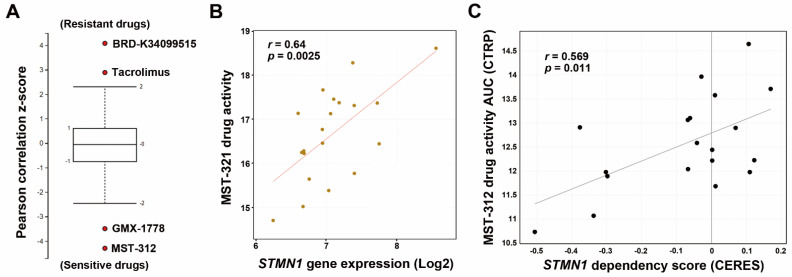
The correlation between STMN1 mRNA expression and MST-312 drug sensitivity in HCC cell lines: (**A**) The drug response profiles correlated with *STMN1* mRNA expression in HCC cell lines were analyzed using the CTRP database; (**B**) A scatter plot for the correlation between *STMN1* mRNA expression and the MST-312 drug activity in HCC cell lines was generated through the CellMinerCDB website. The chemical structure of MST-312; (**C**) A scatter plot for the correlation between the MST-312 drug activity (AUC) and *STMN1* gene dependency was generated through the DepMap website. CERES is a computational method to estimate gene dependency score levels from CRISPR screening. A lower CERES score indicates that a cell has a higher probability of gene dependency. A lower AUC means higher drug activity. CTRP, Cancer Therapeutics Response Portal.

STMN1 has been considered a therapeutic target for cancers [18,19,20]. To identify therapeutic drugs to selectively kill high-STMN1-expressing HCC cells, we mined the CTRP database and found that HCC cells with high *STMN1* mRNA expression were sensitive to MST-312 (a telomerase inhibitor) and GMX-1778 (a competitive inhibitor of nicotinamide phosphoribosyltransferase) but resistant to BRD-K34099515 (unknown function) and Tacrolimus/FK506 (an immunosuppressive drug) (Figure 4A). We focused on investigating the most sensitive drug MST-312 (its chemical structure is shown in the embedded diagram of Figure 4B). A scatter plot showed the positive correlation between *STMN1* mRNA expression and MST-312 drug activity (Figure 4B). To further confirm that the MST-312 drug activity was dependent on STMN1 downregulation, the *STMN1*-CRISPR-knockdown dependency data of HCC cells were obtained from the DepMap website. As shown in Figure 4C, MST-312 drug activity was highly correlated with the *STMN1* dependency in 19 liver cancer cell lines.

**Figure 5:**
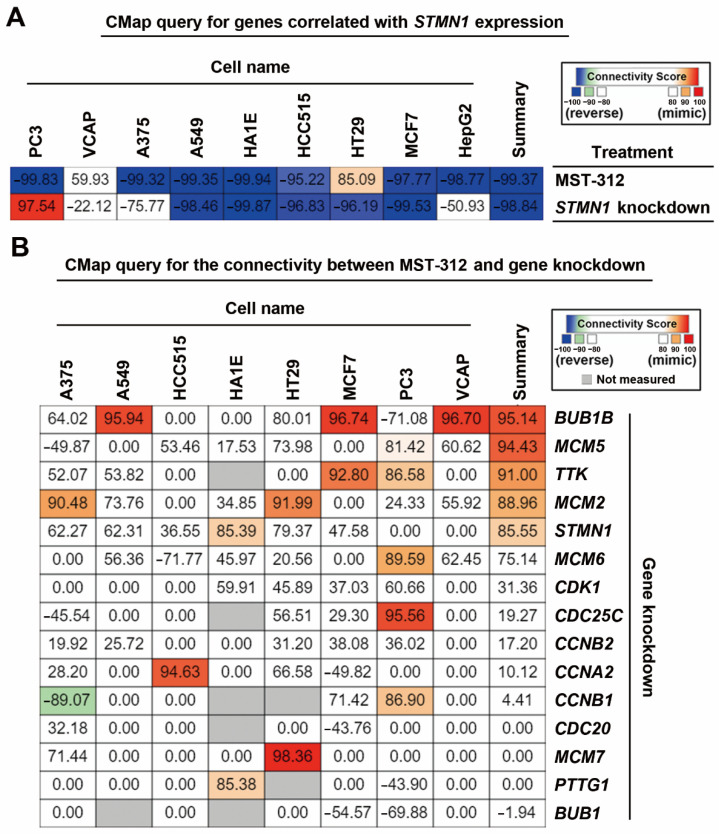
Connectivity mapping for the STMN1-co-expressed genes: (**A**) Connections of *STMN1*-co-expression genes (57 common genes in TCGA-LIHC and Roessler Liver 2 datasets) with the MST-321 and *STMN1* knockdown were analyzed through the CMap website; (**B**) Connections of MST-312 and the selective gene-knockdown signatures were obtained from the CMap website.

According to the above analyses, we hypothesized that the efficacy of MST-312 against high-STMN1-expressing HCC cells may be due to its ability to downregulate and/or inactivate *STMN1* and then reverse *STMN1*-co-expressed gene signature. To demonstrate this possibility, a CMap analysis was performed. The 57 *STMN1*-co-expressed genes were submitted and queried by the CMap database, and then the connection between *STMN1*-co-expressed gene signature and MST-321 was visualized as a heat map (Figure 5A). As expected, the *STMN1*-co-expressed gene signature can be reversed by MST-321 treatment. Interestingly, the *STMN1*-co-expressed gene signature was also reversed by *STMN1*-knockdown (Figure 5A), supporting the dependency of high-*STMN1*-expressing HCC cells on *STMN1* gene expression.

Because the CMap database contains gene signatures from drug-treated or shRNA/cDNA-transfected cancer cell lines [40], it can be used to explore the connections between drugs and genetic knockdown/overexpression. When querying the CMap database for the relationships between MST-312 and knockdown of *STMN1*-co-expressed gene signature, we found that MST-312 treatment was similar to knockdown of *BUB1B*, *MCM2*/*5*/*6*, *TTK*, and *STMN1* (Figure 5B). Therefore, the anticancer potential of MST-321 against high-STMN1-expressing HCC cells may result from its inhibitory effect on DNA replication (MCM2/5/6) and mitosis (BUB1B, TTK, STMN1).

**Figure 6:**
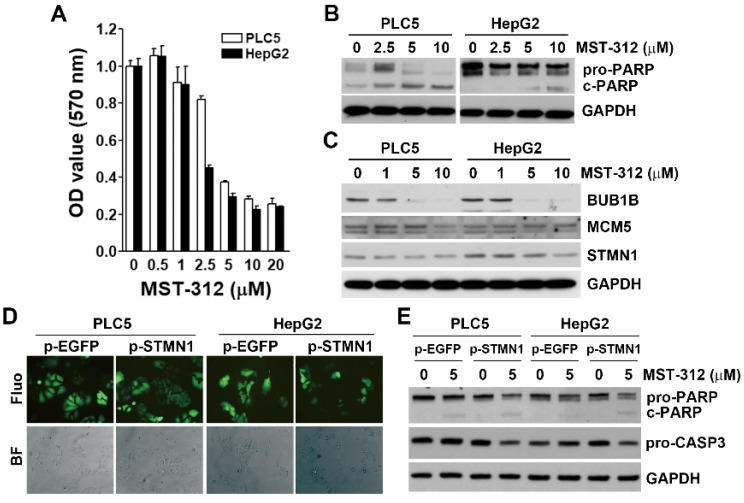
Role of STMN1 in the in vitro anticancer effect of MTS-312: (**A**) PLC5 and HepG2 cells were treated with various doses of MST-312 for 72 h, and then cell viability was determined with Alamar Blue cell viability assay; (**B**) PLC5 and HepG2 cells were treated with the indicated concentrations of MST-312 for 72 h, and protein expression was determined by Western blotting; (**C**) PLC5 and HepG2 cells were treated with various doses of MST-312 for 24 h, and then protein expression was determined by Western blotting; (**D**) PLC5 and HepG2 cells were transfected with pcDNA3-STMN1-EGFP or pcDNA3-EGFP plasmid for 48 h and then selected with 1 mg/mL G418 for at least 3 months. The GFP fluorescence and cell morphology were observed under a fluorescence (Fluo) or bright-field (BF) microscope, respectively; (**E**) STMN1- and EGFP-overexpressing PLC5 and HepG2 cells were treated with 5 μM MST-312 for 72 h, and then protein expression was determined by Western blotting.

To validate our bioinformatics analyses, two HCC cell lines (PLC5 and HepG2) were used. The cell viability assay indicated that MST-312 was an effective anticancer drug against HCC cells (Figure 6A), which were associated with the induction of apoptosis as indicated by the cleavage of PARP1 (Figure 6B). MST-312 indeed inhibited the protein expression of STMN1, BUB1B, and MCM5 (Figure 6C). To further confirm the role of STMN1 in the anticancer activity of MST-312, stable STMN1-overexpressing HCC cells were established (Figure 6D). STMN1 overexpression enhanced the apoptosis-inducing activity of MST-312 as indicated by the cleavages of PARP1 and caspase 3 (Figure 6E), further supporting these findings. Taken together, MST-312 exhibits high anticancer activity against high-STMN1-expressing HCC cells.

## 4. Discussion

We found that STMN1 overexpression in HCC was highly correlated with the overexpression of genes related to cell cycle progression, especially the mitosis stage. As a microtubule-destabilizing protein, fine-tuning of STMN1 activity controls spindle formation during mitosis, and both STMN1 overexpression and downregulation cause the failure of correct mitosis [18]. STMN1 is highly expressed in mitotic hepatocytes and promotes hepatocyte proliferation [46]. Because hepatocytes are the prime cells of origin for malignant transformation during HCC development [47], STMN1 upregulation may occur early during hepatocarcinogenesis.

MST-312 is synthesized as a telomerase inhibitor that can induce telomerase shortening and then inhibit cancer cell growth [48]. Telomerase, which is required for telomere synthesis, consists of telomerase RNA component (TERC) and telomerase reverse transcriptase (TERT). Telomerase activation due to TERT promoter mutation or TERT gene amplification is one of the earliest events during HCC development [49]. The oncogenic addiction of HCC cell lines to TERT was recently identified [50], suggesting that telomerase is an actionable therapeutic target of MST-312 for treating HCC.

The mechanism of STMN1 overexpression in HCC is still largely unclear. Previous studies demonstrate that the E2F transcription factor 1 (E2F1) is involved in *STMN1* transactivation in HCC [51,52]. The E2F transcription factor family (E2F1-E2F8) plays a key role in cell cycle progression and proliferation and also integrates cell cycle progression with DNA repair, replication, and G2/M checkpoint via the coordination of related genes [53]. In addition, the overexpression of the E2F family, especially E2F1, and its oncogenic roles in HCC have been reported [54]. We speculated that the E2F family may contribute to the upregulation of *STMN1* and its co-expressed genes. To confirm this possibility, the 57 *STMN1*-co-expressed genes commonly in TCGA-LIHC and Roessler Liver 2 datasets (Figure S3a) were analyzed for the upstream transcription factors using the WebGestalt online tool. Indeed, these genes contained the potential binding sites for the E2F family (Figure S3b).

Several limitations existed in this study. First, only bioinformatics and in vitro experimental analyses were performed. Further in vivo validation using animal models were required. Second, HepG2 is also referred to as a hepatoblastoma cell line [55], although the original publication indicates that it is derived from a liver hepatocellular carcinoma of a 15-year-old Caucasian male [56]. An additional HCC cell line would increase the data robustness. Third, this study only considered the *STMN1*-co-expressed genes. We could not exclude the potential roles of the genes negatively associated with *STMN1* expression. Fourth, CMap database was employed to predict the effect of MST-312 on reversing the *STMN1*-co-expressed gene signature. Because signatures derived from multiple cancer cell types are usually conversed [40], the CMap database has been widely used for searching for drugs to treat various disease types in addition to cancers [57]. However, cell-type selective gene signatures still exist in 43% of compounds [40], which may be an additional limitation in this study.

In conclusion, this study integrated bioinformatics analysis to explore the role of STMN1 in HCC. We found that STMN1 overexpression is associated with the upregulation of genes related to cell cycle regulation. A telomerase inhibitor MST-312 was found to inhibit high-STMN1-expressing HCC cells via the reversal of the STMN1-co-expressed gene signature. Altogether, this study offers an insight into the therapeutic strategy for STMN1-overexpressing HCC patients.

## Data Availability

The datasets used in this article are publicly available as described in Section 2.

## Supplementary Materials

The following are available online at https://www.mdpi.com/article/10.3390/jpm11050332/s1, Table S1: The STMN1-co-expressed genes in hepatocellular carcinoma, Figure S1: The impact of STMN1 mRNA expression on the overall and disease-free survival of HCC patients, Figure S2: A dot plot showing the results of KEGG pathway enrichment in 4 datasets, Figure S3: Prediction of the upstream transcription regulators for STMN1-co-expressed genes.
