# Supplementary material for: A Bioinformatics Analysis Identifies the Telomerase Inhibitor MST-312 for Treating High-STMN1-Expressing Hepatocellular Carcinoma
Source: J Pers Med. 2021 Apr 22;11(5):332. doi: 10.3390/jpm11050332 (PMC8145764; doi:10.3390/jpm11050332)
Supplement: Supplementary file 1 [file jpm-11-00332-s001.zip › jpm-1175559-supplementary.pdf]

Table S1. The STMN1-co-expressed genes in hepatocellular carcinoma.

TCGA-LIHC		Chen Liver 2		Roessler Liver 2		Wurmbach Liver	
Genes [1]	Correlation	Genes [1]	Correlation	Genes [2]	Correlation	Genes [3]	Correlation
KIFC1	0.829	ZWINT	0.815	BIRC5	0.805	DTL	0.885
KIF2C	0.826	TOP2A	0.802	BUB1B	0.805	KIAA0101	0.885
CDC20	0.825	NUSAP1	0.802	CCNB1	0.805	ZWINT	0.885
RAD54L	0.824	CDKN3	0.802	CCNB2	0.805	RRM2	0.885
ZWINT	0.820	PTTG1	0.802	CDC20	0.805	ASPM	0.863
SPC25	0.819	FOXM1	0.802	CDK1	0.805	SGOL2	0.863
E2F2	0.818	CDK1	0.802	CDK1	0.805	TOP2A	0.863
KIF4A	0.813	UBE2C	0.793	CDK1	0.805	HELLS	0.863
NDC80	0.811	PLK1	0.793	CDKN3	0.805	CEP55	0.863
CDC25C	0.808	CDCA8	0.793	CKS2	0.805	MAD2L1	0.863
CENPM	0.807	CENPF	0.776	DBF4	0.805	CENPK	0.863
CDT1	0.806	NUF2	0.776	DLGAP5	0.805	CCNA2	0.863
NCAPG	0.804	KIAA0101	0.764	DTL	0.805	GINS1	0.863
RAD51	0.804	CENPM	0.764	ECT2	0.805	HMMR	0.863
ASF1B	0.803	MYBL2	0.764	EZH2	0.805	NUSAP1	0.863
PTTG1	0.803	TPX2	0.733	FANC1	0.805	NUSAP1	0.863
TROAP	0.801	TYMS	0.729	FANC1	0.805	CENPE	0.863
OIP5	0.800	CDCA5	0.729	FEN1	0.805	CCNA2	0.863
TACC3	0.799	MAD2L1	0.713	FOXM1	0.805	KIF14	0.863
SKA1	0.796	CEP55	0.713	GINS1	0.805	DEPDC1	0.863
CCNB2	0.795	CENPW	0.713	H2AFX	0.805	DEPDC1	0.863
CDCA8	0.793	KIF23	0.713	HN1	0.805	KIF11	0.863
CDCA3	0.792	BUB1	0.713	KIAA0101	0.805	NUF2	0.863
BIRC5	0.790	C12orf32	0.687	KIF18B	0.805	CCNB2	0.863
CDCA5	0.790	FEN1	0.687	KIF20A	0.805	KIF2C	0.863
CENPA	0.789	UBE2T	0.673	KIF4A	0.805	CDC20	0.863
SKA3	0.789	ARK4A	0.670	KPNA2	0.805	TTK	0.863
CDC45	0.786	TRIP13	0.670	KPNA2	0.805	SGOL2	0.863
CDK1	0.785	NEK2	0.664	MCM2	0.805	CENPA	0.863
FANCI	0.785	NUF2	0.659	MCM3	0.805	BUB1	0.863
TPX2	0.784	NUDT1	0.652	MCM6	0.805	NCAPG	0.863
RFC4	0.782	CDC45	0.652	MELK	0.805	KIF4A	0.863
PCLAF	0.778	E2F1	0.652	NEK2	0.805	ANLN	0.863
NUF2	0.778	MLF1IP	0.644	NUSAP1	0.805	ANLN	0.863
HJURP	0.777	ASF1B	0.644	NUSAP1	0.805	UBE2T	0.863
CENPH	0.774	MCM6	0.644	PBK	0.805	PBK	0.863
GINS1	0.772	IFIT1	0.644	PRC1	0.805	CDK1	0.863
HMGB2	0.770	DTL	0.644	PTTG1	0.805	CDK1	0.863
FAM72B	0.770	FAM189B	0.609	RACGAP1	0.805	CDK1	0.863
ERCC6L	0.768	UBE2T	0.609	RFC4	0.805	NDC80	0.863
TEDC2	0.768	GMNN	0.609	RRM2	0.805	MELK	0.863
MELK	0.767	GMNN	0.609	SMC4	0.805	CDKN3	0.863
CCNB1	0.767	TMEM106C	0.609	SMC4	0.805	CDKN3	0.863
KIF18B	0.765	RRM2	0.609	TOP2A	0.805	PTTG1	0.863
UBE2C	0.765	ATAD2	0.609	TOP2A	0.805	RACGAP1	0.863
PLK1	0.764	ATAD2	0.609	TPX2	0.805	CCNB1	0.863
PKMYT1	0.764	GPC3	0.609	TTK	0.805	NCAPG	0.863
E2F1	0.764			TUBG1	0.805	BUB1B	0.863
DEPDC1	0.763			TYMS	0.805	PRC1	0.863
KIF11	0.763			UBE2C	0.805	DLGAP5	0.863
MCM10	0.763			ZWINT	0.805	NEK2	0.863
LMNB1	0.762			GTSE1	0.804	KIF20A	0.863
TCF19	0.760			HJURP	0.804	BIRC5	0.863
EXO1	0.760			NCAPG	0.804	TOP2A	0.863
PIF1	0.759			NCAPG	0.804	HMMR	0.863
FANCG	0.757			TACC3	0.804	CCDC99	0.852
ORC6	0.756			KNTC1	0.801	CENPW	0.852
MCM2	0.755			MLF1IP	0.801	CKAP5	0.848
NUSAP1	0.755			BUB1	0.792	C12orf48	0.848
RRM2	0.755			CENPA	0.792	NCAPG2	0.848
DLGAP5	0.754			KIF2C	0.792	FANCI	0.848
NEK2	0.753			MAD2L1	0.792	MCM7	0.848
MYBL2	0.753			NDC80	0.792	MCM7	0.848
ORC1	0.753			OIP5	0.792	DTL	0.848
AURKB	0.750			DTYMK	0.78	FANCD2	0.841
WDR62	0.748			MCM4	0.771	FAM54A	0.841
KIF23	0.748			MCM4	0.771	SKA1	0.841

KIF15	0.747			MCM4	0.771	PLK1	0.841
CHAF1B	0.747			PRKDC	0.771	C12orf48	0.841
TOP2A	0.746			AURKA	0.766	CENPH	0.841
PRC1	0.746			WHSC1	0.761	MCM10	0.841
CDKN3	0.746			WHSC1	0.761	C5orf34	0.841
EME1	0.745			CCDC99	0.756	DEPDC18	0.841
UBE2T	0.743			CDC25C	0.756	DEPDC1	0.841
KIF18A	0.743			HMMR	0.756	KIF2C	0.841
FANCD2	0.742			HMMR	0.756	GPSM2	0.841
RNASEH2A	0.742			MKI67	0.752	MKI67	0.841
SGO1	0.741			MKI67	0.752	KIF15	0.841
BUB1	0.741			MCM5	0.75	NCAPH	0.841
CCNA2	0.741			MCM5	0.75	CENPF	0.841
TICRR	0.741			MCM7	0.75	DIAPH3	0.841
EZH2	0.740			MCM7	0.75	CDCA8	0.841
BUB1B	0.740			NCAPG2	0.75	KIF23	0.841
TUBA1B	0.738			RNASEH2A	0.75	CDC25C	0.841
TTK	0.738			CKAP2	0.746	SPC25	0.841
NCAPH	0.738			RFC3	0.746	MKI67	0.841
PHF19	0.737			MSH2	0.744	KIF18A	0.841
TRIP13	0.737			NUP155	0.744	HJURP	0.841
FOXM1	0.734			PRIM1	0.744	MKI67	0.841
KIF20A	0.733			GMNN	0.742	E2F8	0.841
CDC6	0.732			GMPS	0.742	CDCA5	0.841
CENPW	0.731			SAC3D1	0.742	CKAP2L	0.841
CCNF	0.730			SPAG5	0.739	OIP5	0.841
DTL	0.728			CCNA2	0.735	BIRC5	0.841
CEP55	0.728			CCNA2	0.735	MKI67	0.841
C17ORF53	0.727			CENPM	0.733	FOXM1	0.841
UHRF1	0.725			FEN1	0.728	KIF18B	0.841
TRAIP	0.725			POLE2	0.728	CDCA3	0.841
SHCBP1	0.724			SMC2	0.728	CCNB1	0.841
FAM72D	0.723			CDC6	0.722	UBE2C	0.841
MKI67	0.722			CDC6	0.722	NCAPD2	0.836
AUNIP	0.721			CDC7	0.722	TPX2	0.836
CDC25A	0.721			TIPIN	0.722	CENPN	0.823
SGO2	0.721			CEP55	0.715	ASPM	0.823
POLQ	0.719			FBXO5	0.715	AURKB	0.823
RMI2	0.718			KIF11	0.715	SHCBP1	0.823
PARPBP	0.716			KIF15	0.715	TRIP13	0.823
DSN1	0.715			KIF23	0.715	STIL	0.813
H2AFZ	0.715			SPC25	0.715	ORC6L	0.810
CIP2A	0.714			E2F8	0.709	FANCG	0.810
SPDL1	0.712			STIL	0.709	MCM8	0.810
TK1	0.711			AURKB	0.705	KNTC1	0.810
CDCA2	0.710			CDCA3	0.705	UHRF1	0.810
NRM	0.709			TROAP	0.705	CENPL	0.806
MCM7	0.709					UBE2S	0.802
PLK4	0.709					EZH2	0.802
GINS2	0.709					KIF14	0.802
STIL	0.709					RFC4	0.802
FBXO43	0.709					ECT2	0.802
MCM6	0.707					AUKRA	0.802
CENPE	0.705						
MTFR2	0.705						
MAD2L1	0.705						
TYMS	0.704						
DEPDC1B	0.704						
FANCB	0.701						
HMMR	0.700						
HELLS	0.700						
MCM5	0.700						

[1] Only genes with the correlation score more than 0.7 were shown. [2] Only genes with the correlation score more than 0.6 were shown. [3] Only genes with the correlation score more than 0.6 were shown.

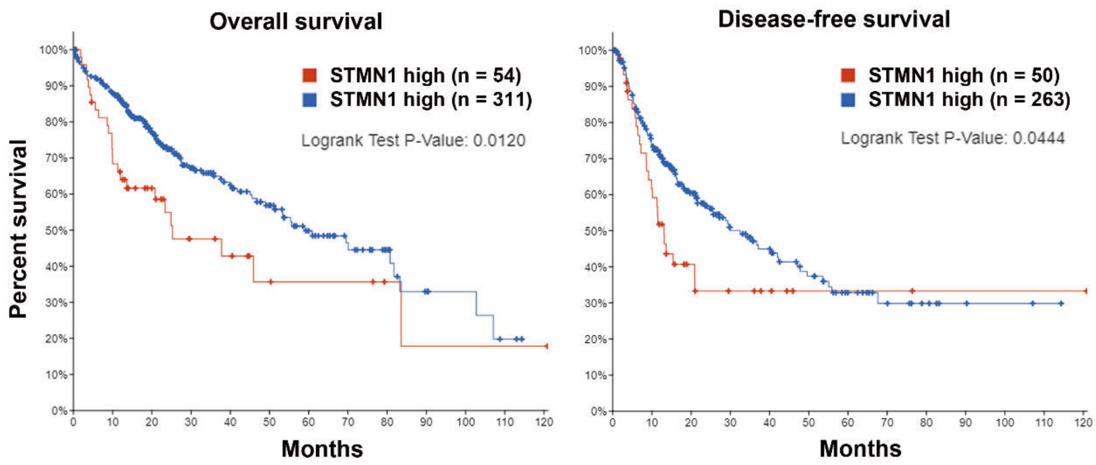

Figure S1. The impact of STMN1 mRNA expression on the overall and disease-free survivals of HCC patients: The Kaplan-Meier survival plots were generated through the cBioPortal website. The group cutoff value was the STMN1 mRNA expression z-score = 1.

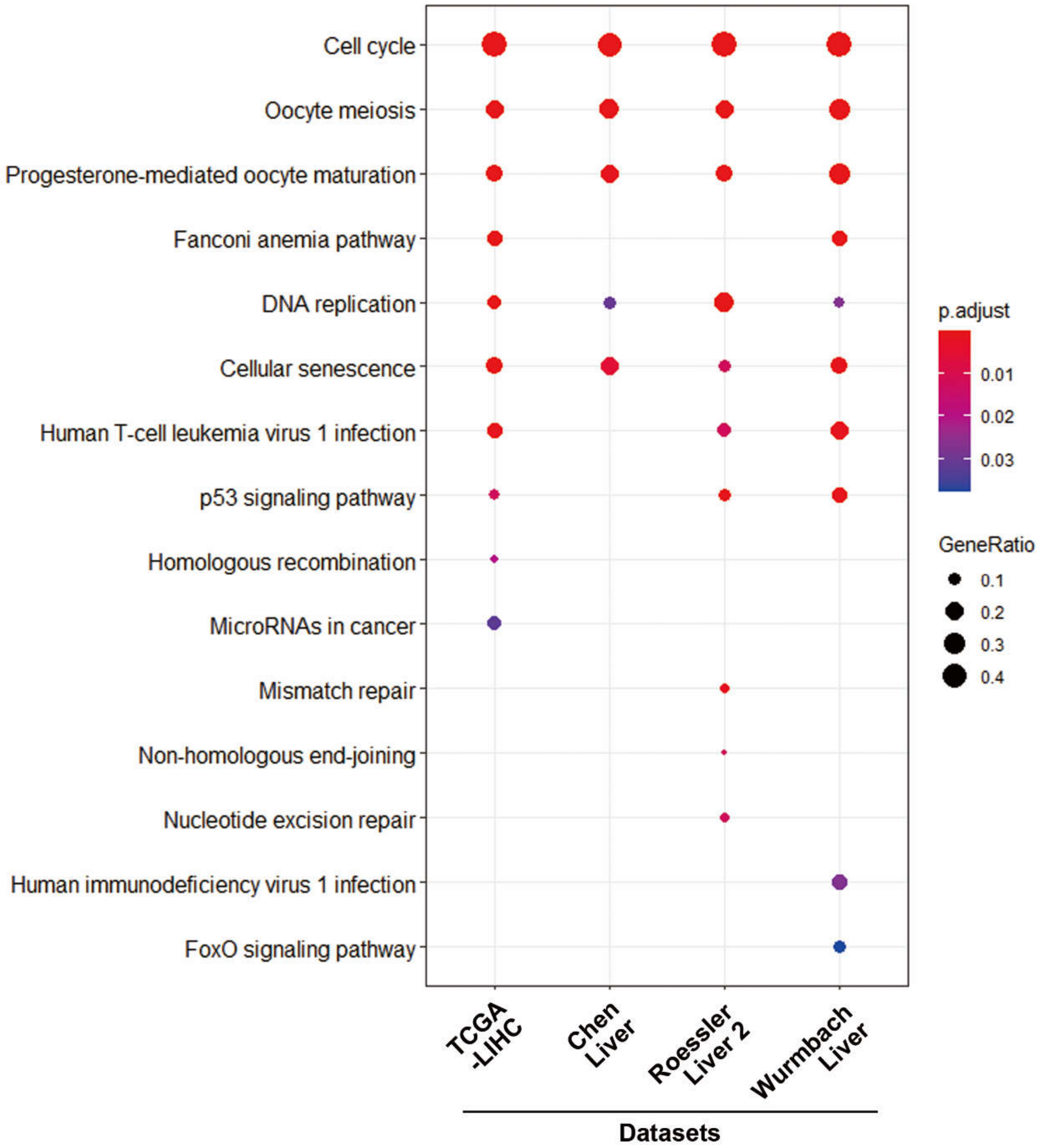

Figure S2. A dot plot showing the results of KEGG pathway enrichment in 4 datasets: STMN1-co-expressed genes in TCGA-LIHC, Chen Liver, Roessler Liver 2, and Wurmbach Liver datasets were analyzed using the clusterProfiler package in R for KEGG pathway enrichment.

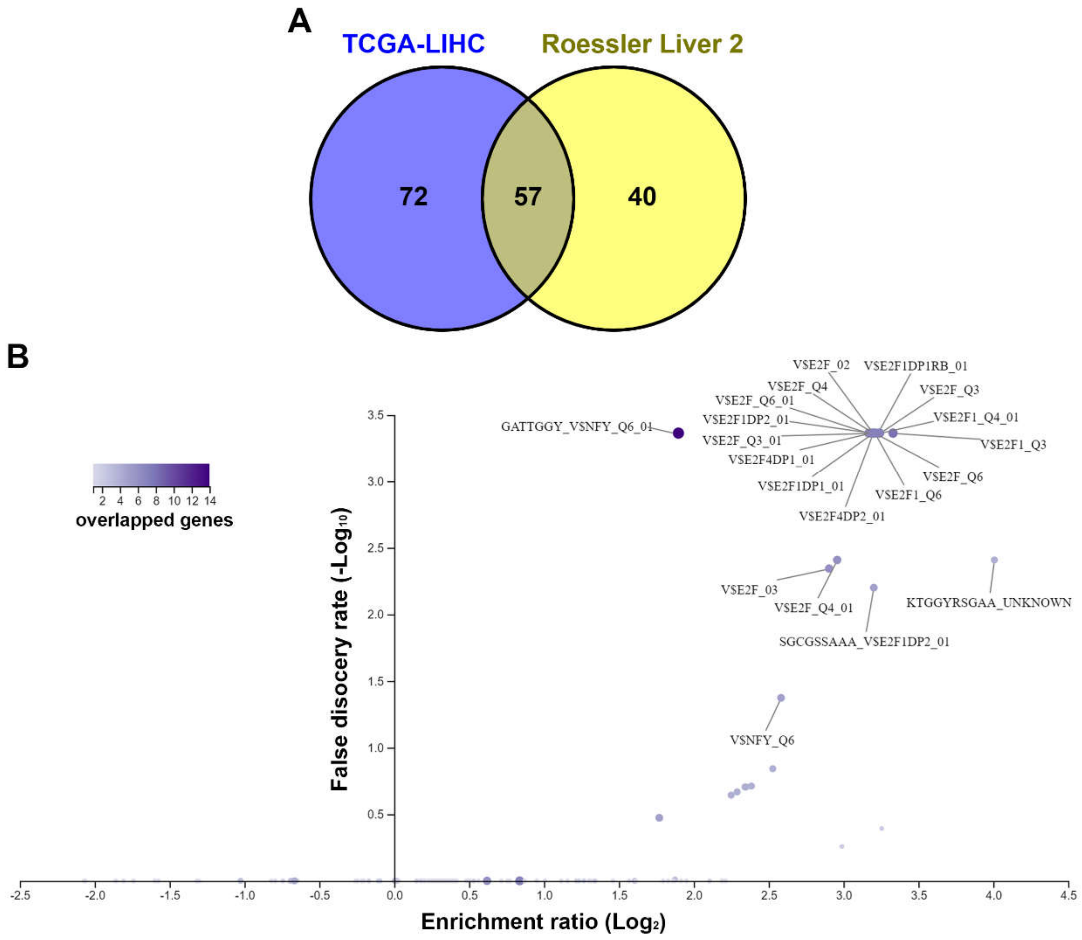

Figure S3. Prediction of the upstream transcription regulators for STMN1-co-expressed genes: (**a**) A Venn diagram showed the overlapped gene numbers for the STMN1-co-expressed genes in the TCGA-LIHC and Roessler Liver 2 datasets. The figure was generated using the VENNY 2.1 (https://bioinfogp.cnb.csic.es/tools/venny/); (**b**) The upstream transcription regulators for 57 common genes were predicted using the WebGestalt online tool.
